# Effect of an Adaptive-Density Filling Structure on the Mechanical Properties of FDM Parts with a Variable Cross-Section

**DOI:** 10.3390/ma15248746

**Published:** 2022-12-07

**Authors:** Jian Liu, Zhou Su, Chenyue Wang, Zhuofei Xu

**Affiliations:** 1 Faculty of Printing, Packaging Engineering and Digital Media Technology, Xi’an University of Technology, Xi’an 710054, China; 2 Zhongchuang Xinhang Technology Co., Ltd., Changzhou 213200, China

**Keywords:** fused deposition modeling, adaptive-density filling structure, mechanical properties

## Abstract

Fused deposition modeling (FDM) technique is one of the most popular additive manufacturing techniques. Infill density is a critical factor influencing the mechanical properties of 3D-printed components using the FDM technique. For irregular components with variable cross-sections, to increase their overall mechanical properties while maintaining a lightweight, it is necessary to enhance the local infill density of the thin part while decreasing the infill density of the thick part. However, most current slicing software can only generate a uniform infill throughout one model to be printed and cannot adaptively create a filling structure with a varying infill density according to the dimensional variation of the cross-section. In the present study, to improve the mechanical properties of irregular components with variable cross-sections, an adaptive-density filling structure was proposed, in which Hilbert curve with the same order was used to fill each slice, i.e., the level of the Hilbert curves in each slice is the same, but the side length of the Hilbert curve decreases with the decreasing size of each slice; hence, the infill density of the smaller cross-section is greater than that of the larger cross-section. The ultimate bearing capacity of printed specimens with the adaptive-density filling structure was evaluated by quasi-static compression, three-point bending, and dynamic compression tests, and the printed specimens with uniform filling structure and the same overall infill density were tested for comparison. The results show that the maximum flexural load, the ultimate compression load, and the maximum impact resistance of the printed specimens with the adaptive-density filling structure were increased by 140%, 47%, and 82%, respectively, compared with their counterparts using the uniform filling structure.

## 1. Introduction

FDM (Fused Deposition Modeling) is a popular 3D printing technique that employs flexible thermoplastic filament injected through a heated nozzle to build components [1]. Before 3D printing, the digital model of the components is converted to STL file format, which is a three-dimensional graphic file format for additive manufacturing and consists of a series of small plane triangle facets that fit the surface of the 3D model. Then the digital model is discretized and layered using 3D model slicing software, and then transformed into a G-code file, which is used to control the nozzle to deposit the melted thermoplastic filament layer by layer according to the predetermined printing path, and finally, form three-dimensional solid parts [2,3]. There are different types and a wide range of feedstock materials for the FDM technique, such as Acrylonitrile Butadiene Styrene (ABS), Polylactic acid (PLA), thermoplastic polyurethane (TPU), polyamide (PA), nylon, polyether ether ketone (PEEK), polyethylene terephthalate glycol (PETG), et al. [4]. With the advantages of simplicity, high material utilization rate, low manufacturing cost, and wide material sources, it is widely used in construction [5,6], automobile [7,8], aerospace [9], medicine [10,11,12], and other fields.

Infill density, i.e., the percentage of infill volume with filament material, is an important parameter in the FDM process affecting the strength of products printed by FDM. It can vary from 0% (hollow part) up to 100% (solid part) [13,14,15,16]. In recent years, the effect of infill density on the mechanical properties of printed parts has been widely studied. Fernandez-Vicente et al. [17] tested the tensile strength of printed parts with different infill densities and found that the variation in infill density significantly affects the tensile strength and stiffness, especially when the infill density is between 20% and 50%. Akhoundi et al. [18] also studied the effect of infill density on the mechanical properties of FDM parts and found that the higher the infill density, the higher the mechanical properties in tensile strength, flexural strength, and elastic modulus. Hikmat et al. [19] considered the effect of seven process parameters (build orientation, raster orientation, nozzle diameter, extruder temperature, infill density, the number of shells, and extruding speed) on the tensile properties of FDM printed parts based on the Taguchi method. According to the results of the signal-to-noise ratio and variance analysis, the build orientation, nozzle diameter, and infill density were statistically significant, and the tensile strength is proportional to the infill density according to a linear regression model. Shafaat et al. [20] investigated the effect of infill density and layer thickness on the mechanical properties of ABS parts by tensile test and found that ultimate tensile strength and elastic modulus increase with increasing infill density. Ivorra-Martinez Juan et al. [21] printed PLA parts with different infill densities and reported that the elastic modulus increased with the increasing infill density. Porter et al. [22] conducted three-point flexural, free vibration, and buckling tests on slender structural members manufactured by FDM and found that the flexural rigidity varied linearly with the infill density (0, 5, 10, 15, 20, 30, 50, 70, and 100%), but decreased rapidly below 10% infill, and the optimal infill density to maximize the specific flexural rigidity was between 10% and 20%. Torres et al. [23] studied the effects of layer thickness, infill density, and heat treatment time at 100 °C on the shear strength of PLA parts. According to the results of the Taguchi test and Analysis of Variance [24], the infill density has a certain effect on the 0.2% shear yield strength, and the ultimate shear strength rises with increasing infill densities. Naik M et al. [25] conducted impact tests on bio-inspired 3D lightweight structures printed in flat and on-edge orientation with infill densities of 20%, 35%, 50%, 65%, 80%, and 100%, and the results showed that the test specimen with 20% infill density printed in on-edge orientation has the highest impact strength.

The strength of a part generally increases when increasing the amount of the material inside the part. However, as the infill density increases, printing time and model weight increase. Gradient infill is a promising method to control the mechanical properties of printed components across a wide range while maintaining the lightness of the structure [26]. Maszybrocka et al. [27] proposed a radial gradient infill method for a cylindrical sample, in which the cylindrical sample was divided into several hollow cylinders and a thin cylinder as the core of the sample, after being filled with increasing infill density from the outer layer to the core cylinder, the elements were assembled again to obtain a variable filling of the model. However, there is little literature about the variable filling for irregular components with non-uniform cross-sections, in which the zone with a small cross-section is generally vulnerable and need higher infill density.

In the present study, an adaptive-density filling structure for a model with a variable section was proposed, which makes the infill density adjustable according to the size of the slice at different heights of the model. Based on the G-code file (recording slicing parameters, printing parameters, tool path, et. al) generated by a self-developed 3D printing pre-processing software, quadrangular specimens with gradually reduced cross-sections were built by FDM technology. The mechanical properties of the adaptive-density-filled specimens and uniformly filled specimens were compared using quasi-static compression, three-point bending, and dynamic compression tests.

## 2. Experimental Procedure

### 2.1. Design and Fabrication of Testing Specimens

**Figure 1:**
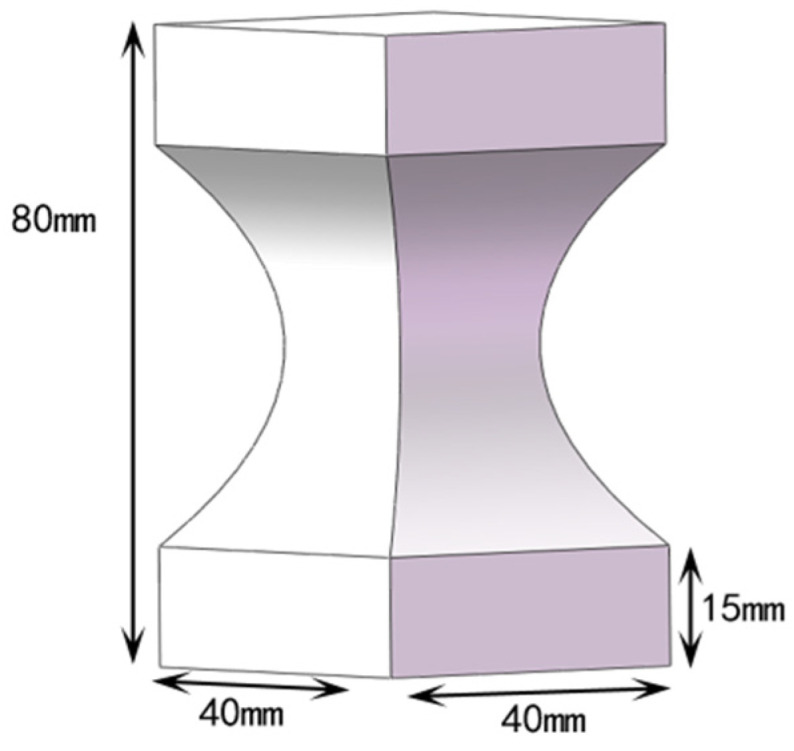
The quadrangular model with a gradually reduced cross-section.

A quadrangular model with a gradually reduced cross-section was designed as a test model using SolidWorks 2016, as shown in Figure 1. The total height of the test model is 80 mm, and the upper and lower ends are cuboids of 40 × 40 × 15 mm^3^. The cross-sectional area between the two cuboids gradually shrinks symmetrically from both ends to the middle, and the cross-section is the smallest at half its height, which is a square of 20 × 20 mm^2^. The quadrangular model is partially filled, and the top and bottom of the model are not covered with a shell to observe the inner infill structure conveniently.

**Figure 2:**
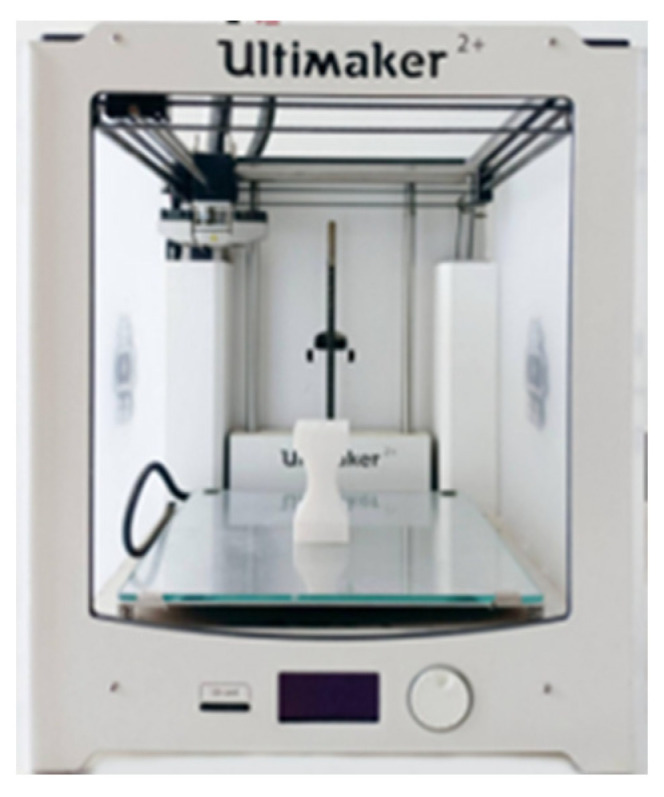
Ultimaker^2+^ printer.

The test specimens were fabricated in a Ultimaker^2+^ printer [28], as shown in Figure 2, which provides a maximum build volume of 223 × 223 × 205 mm^3^. The nozzle of the printer moves in the x and y direction while the build platform moves in the z-direction. The available nozzles are 0.25, 0.4, 0.6, and 0.8 mm in diameter, and the maximum heating temperature can reach 260 °C. The platform temperature is 20–110 °C, the layer resolution is 20–200 μm, and the printing speed is 30–300 mm/s. The printer mainly supports the printing of PLA and ABS, which have been the primary filament materials since the beginning of FDM technology. PLA is an aliphatic thermoplastic polymer that is obtained from raw materials of natural origin, such as cornmeal. Due to its non-toxicity, biodegradability, and biocompatibility, PLA can be used in many industries as well as in medicine in biomedical applications. ABS is a thermoplastic polymer that is extremely resistant to impact, abrasion, and chemical elements [29]. The material used for manufacturing the test specimens was PLA and ABS filament of 3 mm diameter produced by Jingtian Century Technology Co., Ltd. (Zhuhai, China). The density of the PLA filament is 1.24 ± 0.05 g/cm^3^, and its ultimate tensile strength is 60 MPa. The ABS filament has a density of 1.04 ± 0.2 g/cm^3^, its ultimate tensile strength is 72–90 MPa, the flexural strength is 96–120 MPa, and the compressive strength is 96 MPa. All printing parameters used in this study are summarized in Table 1.

**Figure 3:**
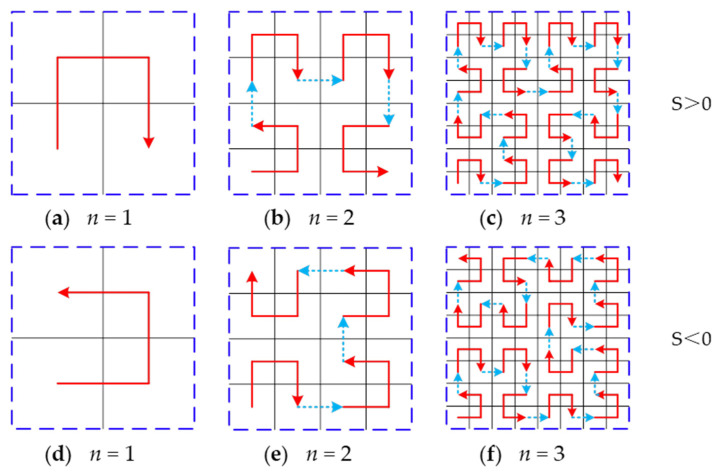
One to three orders of Hilbert curves with movement direction, (**a**,**d**) first-order Hilbert curve, (**b**,**e**) second-order Hilbert curve, (**c**,**f**) third-order Hilbert curve, the blue dash-dotted line denotes the magnified square, and the red arrow denotes the connecting order of grid center.

Hilbert curve was selected as the infill pattern, which is a continuous space-filling fractal curve. Due to its continuity, the start-stop frequency of the motor in the 3D printer can be reduced; thus, the printing efficiency can be improved. To realize the adaptive density filling structure, a pre-processing software for 3D printing was developed with C++ and Visual Studio2019 as the development language and development tool, respectively, which not only has similar functions to Ultimaker Cura 4.8.0 (a slicing software) [30], such as model reading, display, geometric transformation, and slicing but also can adaptively change the infill density according to the size of the sliced contour. The main implementation steps of the adaptive density filling are as follows [31]:

1. The quadrangular model is transferred to the self-developed 3D printing pre-processing software and sliced into numerous layers of equal thickness. The intersection points between triangular facets approximating the surfaces of the model and each slice are connected sequentially, and the obtained cross-sectional contour information of each slice is stored.
2. A bounding box for each slice is calculated and a higher dimension side of the bounding box is taken as the side of the minimum square (*L*) for which the Hilbert curve is to be generated by recursion [32,33]. To ensure the Hilbert curve contacts with the outer shell, the side of the minimum square was magnified by 2*^n^*/(2*^n^* – 1) times, where *n* denotes the order of the Hilbert curve.
3. Hilbert curves of specified order with the same level are generated within the magnified square layer by layer.
4. The Hilbert curve outside the contour is trimmed to fit the contour, and the trimmed Hilbert curve coordinates are stored. The generation strategy of the Hilbert curve is shown in Figure 3 [34]. The magnified square of each slice contour is divided into 2 × 2 square grids, and the centers of the four-square grids are connected in sequence from the south-west corner in a clockwise (denoted by S > 0) or counterclockwise (denoted by S < 0) direction, and cup-shaped first-order Hilbert curves facing south and west are generated, respectively, as shown in Figure 3a,d. If the magnified square is divided into 2^2^ × 2^2^ square grids, and draw a single curve with every center of the square grids in certain rules until the curve fulfills the whole plane, a second-order Hilbert curve is generated, as shown in Figure 3b,e. In the same way, a third-order Hilbert curve is generated by connecting every center of the square grids with 2^3^ × 2^3^ sections, as shown in Figure 3c,f.

**Figure 4:**
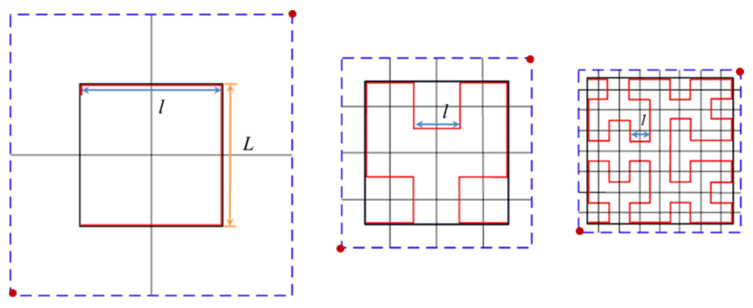
The side lengths of Hilbert curves with different orders (*n* = 1, 2, 3 from left to right), the black solid line, and the blue dash-dotted line denote the original and magnified square, respectively, and the red line denotes the generated Hilbert curves.

To enable the infill density of the filling curve to change according to the cross-sectional area, the order of Hilbert curve *n* is taken as a fixed value. The cross-section is filled layer by layer, and the side length *l* equals *L*/(2*^n^* – 1), when the *n* equals 1, 2, 3, the side length *l* equals *L*, *L*/3, and *L*/7, respectively, as shown in Figure 4.

**Figure 5:**
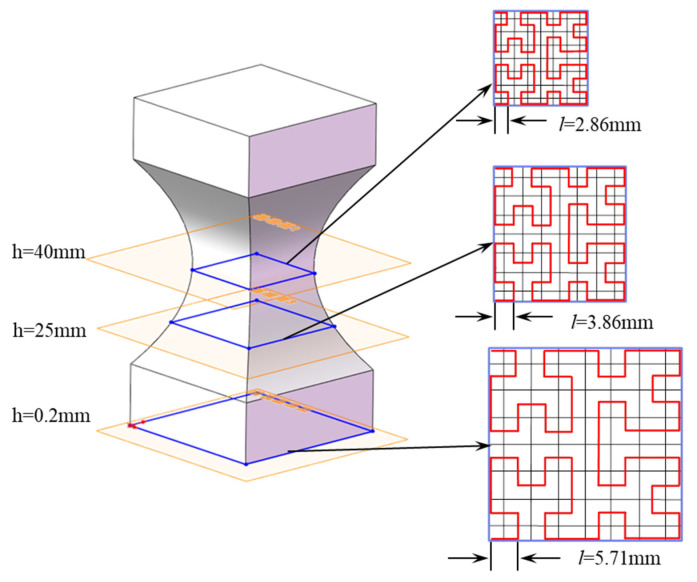
The adaptive density filling structure with the Hilbert curve with the same order.

When the order of Hilbert curve *n* takes 2, each slice was subdivided thrice, and the infill patterns within each slice at various heights are similar, as shown in Figure 5, but the side length of the Hilbert curves decreases with decreasing cross-sectional area. When the heights of the model are 0, 25, and 40 mm, the corresponding cross-section gradually decreases, and the theoretical value of the side lengths of the Hilbert curves are 5.71, 3.86, and 2.86 mm, respectively.

### 2.2. Characterization of the Infill Structure and Mechanical Properties

To characterize and evaluate the infill effect of the adaptive-density filling structure, the infill densities at different heights of the printed specimens were determined by image processing and analysis. For the adaptive-density filling structure, the infill densities of the test specimen are different layer by layer. The overall infill density of test specimens with the adaptive-density filling structure was determined by

(1) $$\rho \;\approx \frac{V_{F}}{V_{T}}\times 100\%=\frac{{\pi R}^{2}L_{F}}{V_{T}}\times 100\%$$

where $V_{F}$, $V_{T}$ and R represents the total amount of the filament used for infill (mm^3^), the apparent volume of the test specimen (mm^3^), and the filament radius (mm), respectively. $L_{F}$ denotes the length of the filament used for infill (mm), which can be read from G-code. After calculation, the overall infill density of the test specimen is about 15%. Contrast models with the same apparent size were imported into Slic3r software to slice and filled with a uniform Hilbert curve of 15% infill density. To ensure the accuracy and repeatability of the test results, three test specimens with adaptive-density filling structures and three test specimens with uniform filling structures were fabricated with the PLA filament and denoted as A1, A2, A3, and S1, S2, and S3, respectively.

To investigate the variation of infill density in the cross-sections of the quadrangular model with gradually reduced cross-section, several PLA specimens were incompletely printed until reaching certain heights. The exposed cross-sections of the partially printed specimens with different heights were captured by a digital camera, and the obtained digital photographs with a DPI (points per inch) of 96 were subjected to grayscale processing in PhotoShop CC 2019 to obtain a black and white image, where the black part is the Hilbert curve. The image pixel values of the Hilbert curve and the entire filled area (including no contour) were extracted. The infill density equals the ratio of the image pixel value of the Hilbert curve to that of the entire filled area.

**Figure 6:**
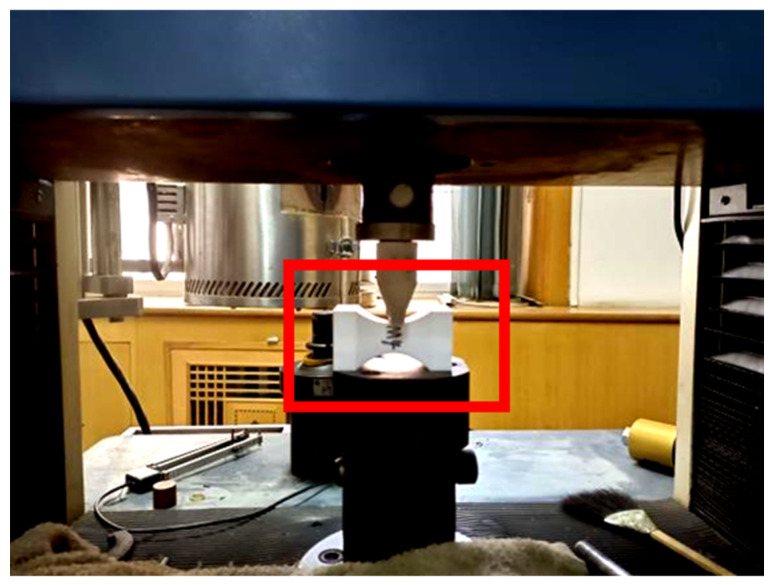
The photograph of the three-point bending test.

To investigate the effect of the adaptive-density filling structure on the load-bearing capacity of the printed components, quasi-static compression tests, three-point flexural tests, and dynamic compression tests were carried out. The compression tests and three-point flexural tests were performed on an HT-2402/100KN universal testing machine (produced by Taiwan Hongda Instrument Co., Ltd., Taiwan, China) at a constant crosshead speed of 2 mm/min and 1 mm/min, respectively, and the load-displacement data in the test process were recorded in real-time. In the three-point bending test, the test specimen was placed horizontally, and a vertical load was applied to the middle of the specimen, as shown in Figure 6.

**Figure 7:**
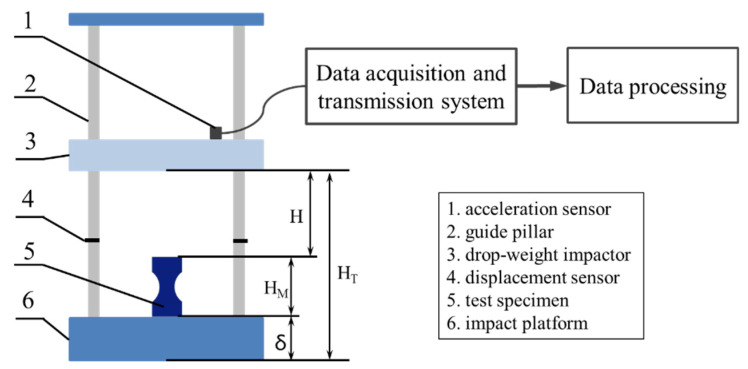
Schematic diagram of the dynamic compression tester.

The dynamic compression tests were carried out on a DY-3 dynamic compression tester (see Figure 7) produced by Xi’an Jiesheng Electronic Technology Co., Ltd. (Xi’an, China). The dynamic compression tester is mainly composed of a drop-weight impactor, acceleration sensor attached to the impactor, guide pillar, displacement sensor, and impact platform. The minimum drop height recorded by the sensor is 20 cm, the weight of the impactor is 7 kg, and its drop process before contacting the test specimen is in free fall. The drop height, impact energy, and load in the impact process are calculated as follows:

(2) $${H=H}_{T}\;{-\;\delta \;-\;H}_{M}$$

(3) E=mgH

(4) F=mag

where H, $H_{T}$, $H_{M}$, and *δ* denotes drop height, the height read from the test machine scale, the height of the test specimen, and the height of the impact platform with a fixed value of 27 cm. All measurement units are cm. In Equation (3), *E* is the impact energy with a unit of J, and *m* is the impactor weight with a unit of kg. In Equation (4), *F* represents the load in the impact process with a unit of N, *a* is the ratio of acceleration recorded by the acceleration sensor to gravitational acceleration *g* (taking 9.8 m/s^2^).

## 3. Results and Discussion

### 3.1. Infill Density Distribution

**Figure 8:**
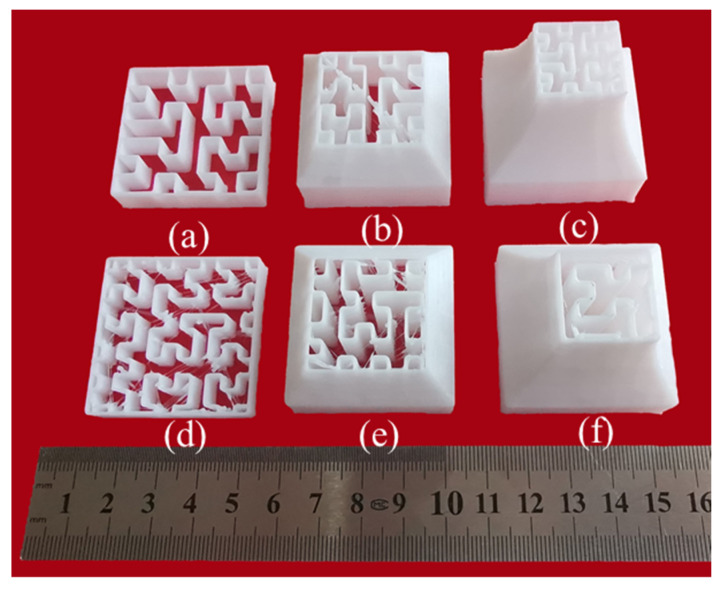
The cross-sections of the incomplete specimens with different heights: 10 mm (**a**), 25 mm (**b**), 40 mm (**c**) adaptive-density filling structure, 10 mm (**d**), 25 mm (**e**), 40 mm (**f**), uniform filling structure.

The specimens with adaptive-density filling structures and uniform filling structures were incompletely printed with a height of 10 mm, 25 mm, and 40 mm, respectively, and the side length of the Hilbert curves was measured using a digital caliper with an accuracy of 0.01 mm. The corresponding cross-section of the incompletely printed samples with different heights is shown in Figure 8. It was found that the measured average side length of the Hilbert curves for the uniformly filling structure is 3.78 ± 0.01 mm in the cross-sections at any height, whereas the average side length of the Hilbert curves for the adaptive-density filling structure is 5.51 ± 0.02 mm, 3.79 ± 0.03 mm, and 2.72 ± 0.04 mm, respectively, indicating that the side length of the Hilbert curve decreases with the decreasing cross-section for the adaptive-density filling structure.

**Figure 9:**
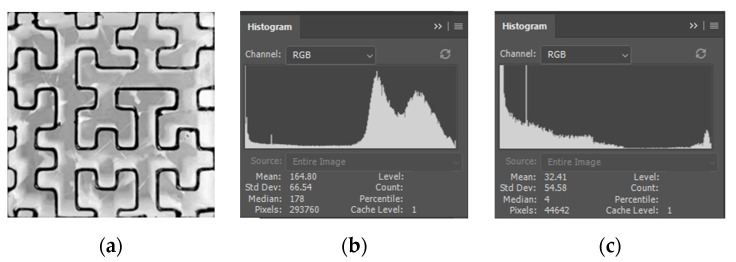
Black and white image of cross-section except for contour (**a**); gray level histogram of the entire filled area (**b**); gray histogram of the black filling curve (**c**).

To quantitatively determine the infill density of various cross-sections at different heights, specimens with adaptive-density filling structures and uniform filling structures were incompletely printed with a height of 15 mm, 25 mm, and 35 mm, respectively, and the black and white images obtained after grayscale processing is shown in Figure 9a, in which the black part is the filling curve. Figure 9b,c exhibit the gray histogram of the entire filled area and the black filling curve, respectively. The pixel values of the black filling curve, the entire filled area, and their ratio are listed in Table 2, and the pixel ratio can be approximately regarded as the infill density.

**Figure 10:**
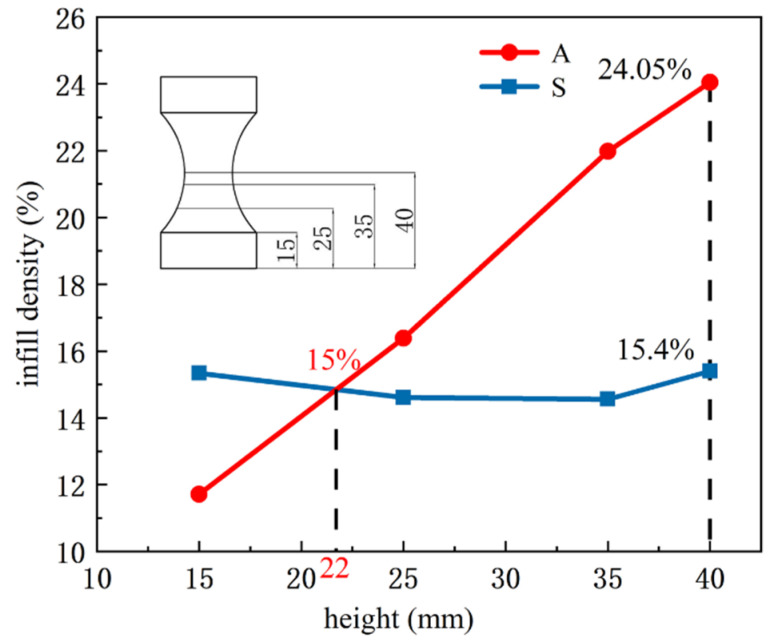
Infill density at a different height (S denotes uniform filling structure, and A denotes adaptive-density filling structure).

Figure 10 shows the infill density curve of the incompletely printed specimens with different heights (15–40 mm). As can be seen from Figure 10, the infill density of each cross-section is about 15% for the specimen with the uniform filling structure, and the cross-sectional infill density of the adaptive-density filling structure increases with height. When the height is less than 22 mm, the variable-density-filled specimen is less than the uniformly filled one in infill density. When the height is greater than 22 mm, the infill density of the former is greater than the latter. The infill density at the height of 40 mm reaches the maximum value of 24.05%, which is 8.65% higher than that of the uniformly filled specimen.

It was evident that an adaptive-density filling structure based on the Hilbert curve was generated for 3D-printed components with variable sections, and each slice was filled with a Hilbert curve at the same level, but the side lengths of the Hilbert curve is different, hence a variable density infill in one model was realized. For the printed specimens with an adaptive-density filling structure, the smaller the slice size, the denser the Hilbert curve seems, and the greater the infill density is consequently. The strengthening effect of the adaptive-density filling structure was evaluated in the subsequent section.

### 3.2. Mechanical Properties

**Figure 11:**
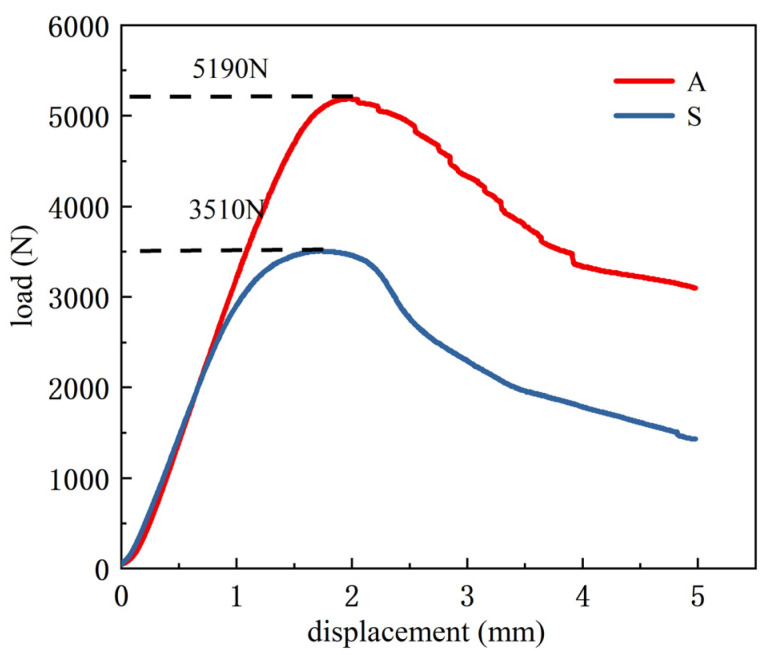
Compression load-displacement curve (S denotes uniform filling structure, and A denotes adaptive-density filling structure).

Figure 11 depicts the load-displacement curves of the specimens with two infill structures. It can be seen from Figure 11 that the load increases rapidly with the increase in compression displacement, after reaching the peak, the load decreases gradually. The peak loads of curve S and curve A are 3510 N and 5190 N, and the compression displacements corresponding to the peak load are 1.68 mm and 2.0 mm, respectively. The load-bearing capacity of the adaptive-density-filled specimen is 1680 N higher than that of the uniformly filled specimen and improved by about 47%. The failure time of the adaptive-density-filled specimen is also later than that of the uniformly filled specimen, which confirms that the adaptive-density filling structure is beneficial to improving the overall mechanical properties of the printed specimen.

**Figure 12:**
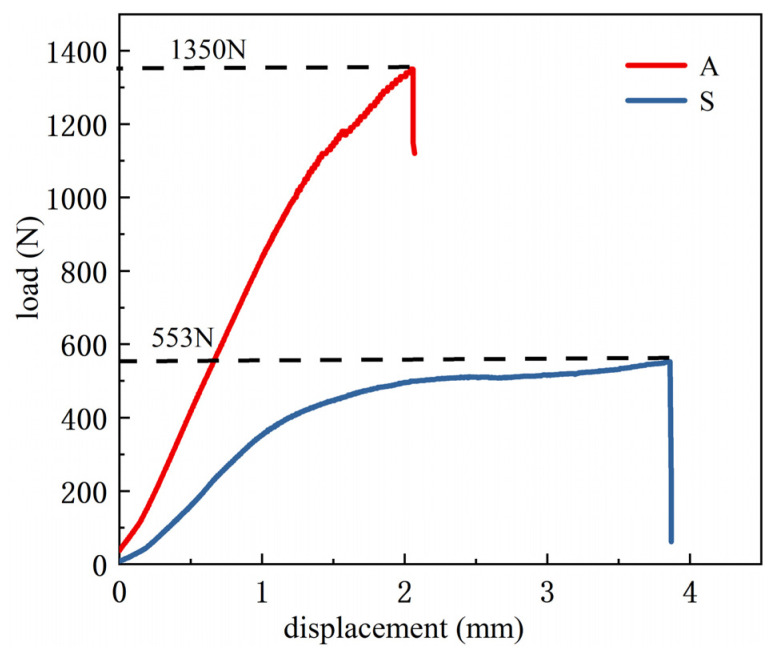
Flexural load-displacement curve (S denotes uniform filling structure, and A denotes adaptive-density filling structure).

Figure 12 presents the load-displacement curve recorded during the three-point bending test. As can be seen from Figure 12, the peak load of the uniformly filled specimen is 553 N, and the displacement to fracture is 3.86 mm. The peak load of the adaptive-density-filled specimen is 1350 N, and the displacement to fracture is 2.06 mm. Although the fracture time of the adaptive-density-filled specimen is earlier, the ultimate flexural load is increased by nearly 1.4 times, indicating that the overall flexural bearing capacity was improved by adjusting the infill density distribution within the specimen. The improved flexural resistance may be due to the following aspects: firstly, the infill density of the middle part was increased, so its flexural resistance was enhanced. In addition, small traveling distances of the nozzle due to the small side length are beneficial to maintain the high temperature of rasters, promoting the bonding between the rasters and deposited layers below, and thereby improving the load-bearing capacity of the printed components [35].

It was found that the test specimens of PLA were crushed during the dynamic compression tests due to their low impact resistance and low strength, and it may be due to the intrinsic brittleness of PLA materials and the low infill density of the test specimens. Hence, the test specimens for the dynamic compression tests were printed again with ABS filament, which has higher toughness and impact resistance. The dynamic compression tests were carried out under drop heights of 20 cm, 22 cm, and 24 cm, and the main test parameters are listed in Table 3.

**Figure 13:**
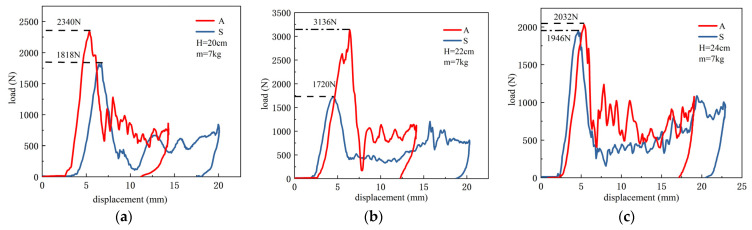
Load–displacement curve during the dynamic compression tests at different drop heights (*H*), *H* = 20 cm (**a**); *H* = 22 cm (**b**); *H* = 24 cm (**c**), (S denotes uniformly filled specimen, and A denotes adaptive-density-filled specimen).

Figure 13 illustrates the load-displacement curves recorded during the dynamic compression tests under a drop height of 20 cm, 22 cm, and 24 cm, respectively. In the process of drop impact, the three groups of curves exhibit a similar trend, when the impactor contacts the test specimen during falling, the impact load increases rapidly to a peak, and then decreases sharply to a certain value, followed by fluctuation with increasing displacement, a similar phenomenon was also reported in Andrew’s study [36], which may be due to the gradual collapse of the filling structure. Under three drop heights, the ultimate load of the adaptive-density-filled specimen is greater than the uniformly filled specimen, and the gap of the maximum ultimate load between the two filling structures is the greatest when the drop height is 22 cm, the former is 82% greater than the latter.

From the testing results of the uniaxial compression, three-point flexural, and dynamic compression tests, it can be concluded that under the same overall infill density, the printed specimens with the adaptive-density filling structure exhibited greater load-bearing capacity compared with the specimens with a uniform filling structure. The flexural bearing capacity, the compression bearing capacity, and the maximum dynamic impact resistance of the former were increased by 140%, 47%, and 82%, respectively, compared with the latter. The overall load-bearing capacity of the printed specimens can be improved by utilizing the adaptive-density filling structure by adjusting the infill density distribution throughout the model.

## 4. Conclusions

An adaptive-density filling method was proposed in the present study, by which a variable-density filling structure in one 3D-printed part can be generated according to the dimensional variation of its cross-section. It was confirmed experimentally that the adaptive-density filling method can contribute to a higher overall bearing capacity under a statistic compression load, flexural load, and drop impact load. The present study provides new insight into improving the mechanical properties of 3D-printed parts while maintaining a lightweight. However, the strengthening mechanism remains unknown, and the local infill density cannot be controlled quantitatively. In our future study, finite element simulation will be introduced to reveal the stress distribution, and the relationship between infill density and the bearing capacity, thereby customizing the adaptive-density filling structure for a certain component to be printed.

## Figures and Tables

**Table 1:** The process parameters used in printing the test specimens.

Parameters	Values		Units
	PLA	ABS	
Infill density	15		%
Printing speed	30		mm/s
Bed temperature	60	80	°C
Nozzle temperature	210	250	°C
Layer thickness	0.2		mm
Nozzle diameter	0.8		mm
Number of Wall	1		–
Filament diameter	3		mm

**Table 2:** Pixel ratio based on the gray histogram.

	Height	15 mm	25 mm	35 mm	40 mm
Pixels					
$S_{total}$		293,760	1,918,224	158,800	155,610
$S_{black}$		44,642	280,214	23,116	23,960
$S_{black}/S_{total}$		15.20%	14.61%	14.56%	15.40%
$A_{total}$		3,452,064	412,804	187,050	124,236
$A_{black}$		404,392	67,653	41,123	29,877
$A_{black}/A_{total}$		11.71%	16.39%	21.98%	24.05%

**Table 3:** The main parameters of the dynamic compression tests.

Drop Height/cm.	Impactor Gravity/kg	Impact Energy/J
20	7	13.72
22	7	15.09
24	7	16.46

## Data Availability

Not applicable.
